# The Primates of Gorongosa National Park, Mozambique

**DOI:** 10.1002/ajpa.70143

**Published:** 2025-10-28

**Authors:** Susana Carvalho, Robert L. Anemone, João d’Oliveira Coelho, René Bobe

**Affiliations:** ^1^ Department of Science Gorongosa National Park Sofala Mozambique; ^2^ CIBIO, Centro de Investigação Em Biodiversidade e Recursos Genéticos Vairão Portugal; ^3^ Interdisciplinary Center for Archaeology and Evolution of Human Behaviour ‐ ICArEHB Universidade Do Algarve Faro Portugal; ^4^ School of Anthropology University of Oxford Oxford UK; ^5^ Department of Anthropology University of North Carolina at Greensboro Greensboro North Carolina USA

**Keywords:** *Cercopithecus*, *Chlorocebus*, Galagidae, mosaic environments, *Papio*

## Abstract

This contribution is an introduction to and synthesis of the special issue of the AJBA on *Primate Adaptations in a Highly Seasonal and Heterogeneous African Ecosystem*. The eight research papers in this special issue provide the first compilation of primatological research to emerge from Gorongosa National Park and represent a major landmark in the development of primatology as a science in Mozambique. Primatological field studies in the park were initiated in 2016 under the umbrella of the Paleo‐Primate Project Gorongosa with the aim of exploring the deep time evolutionary history of the Gorongosa ecosystem and establishing a long‐term primatological field research program. This initiative has resulted in the training of a new generation of primatologists, including the first from Mozambique. The papers in this volume focus on the behavior, ecology, adaptations, and genomics of baboons and vervet monkeys, and set the stage for the study of other primates in Gorongosa, including samango monkeys and nocturnal strepsirrhines. The environmental characteristics of the Gorongosa ecosystem, with major rivers and lakes in a dynamic mosaic of forests, woodlands, wetlands, and grasslands, and rich biodiversity, make Gorongosa a suitable analog for the environments in which early hominins are thought to have evolved. This special issue is dedicated to the memory of our dear friend and colleague Dr. Marc Stalmans, who was the Director of Science of Gorongosa National Park from 2012 to 2025.

## 1 Introduction

Gorongosa National Park (GNP) in central Mozambique is becoming a powerful workshop for the study of ecology and evolution in a dynamic tropical terrestrial ecosystem (Campbell‐Staton et al. 2021; Grabowski et al. 2024; Pringle 2017; Stalmans et al. 2019). Researchers working in the park are also leading the establishment of primatology as an emerging science in Mozambique (Beardmore‐Herd et al. 2025; Biro et al. 2025; Farassi et al. 2025; Hammond et al. 2022; Lewis‐Bevan et al. 2025a, 2025b). The park has a high abundance of nonhuman primates (hereafter, primates), including baboons, vervets, samango monkeys, and two species of nocturnal strepsirrhines (galagos) (Stalmans and Peel 2024; Tinley 1977). The latest aerial survey of GNP in November 2024 covered 61% of the park's surface area and documented 229 baboon troops (Stalmans and Peel 2024). This latest survey also counted more than 110,000 individuals among the larger vertebrate species (> 5 kg), with a high diversity of ungulates and growing populations of carnivores, underscoring the ecological richness of the ecosystem and its value for primatological research in Africa.

**FIGURE 1:**
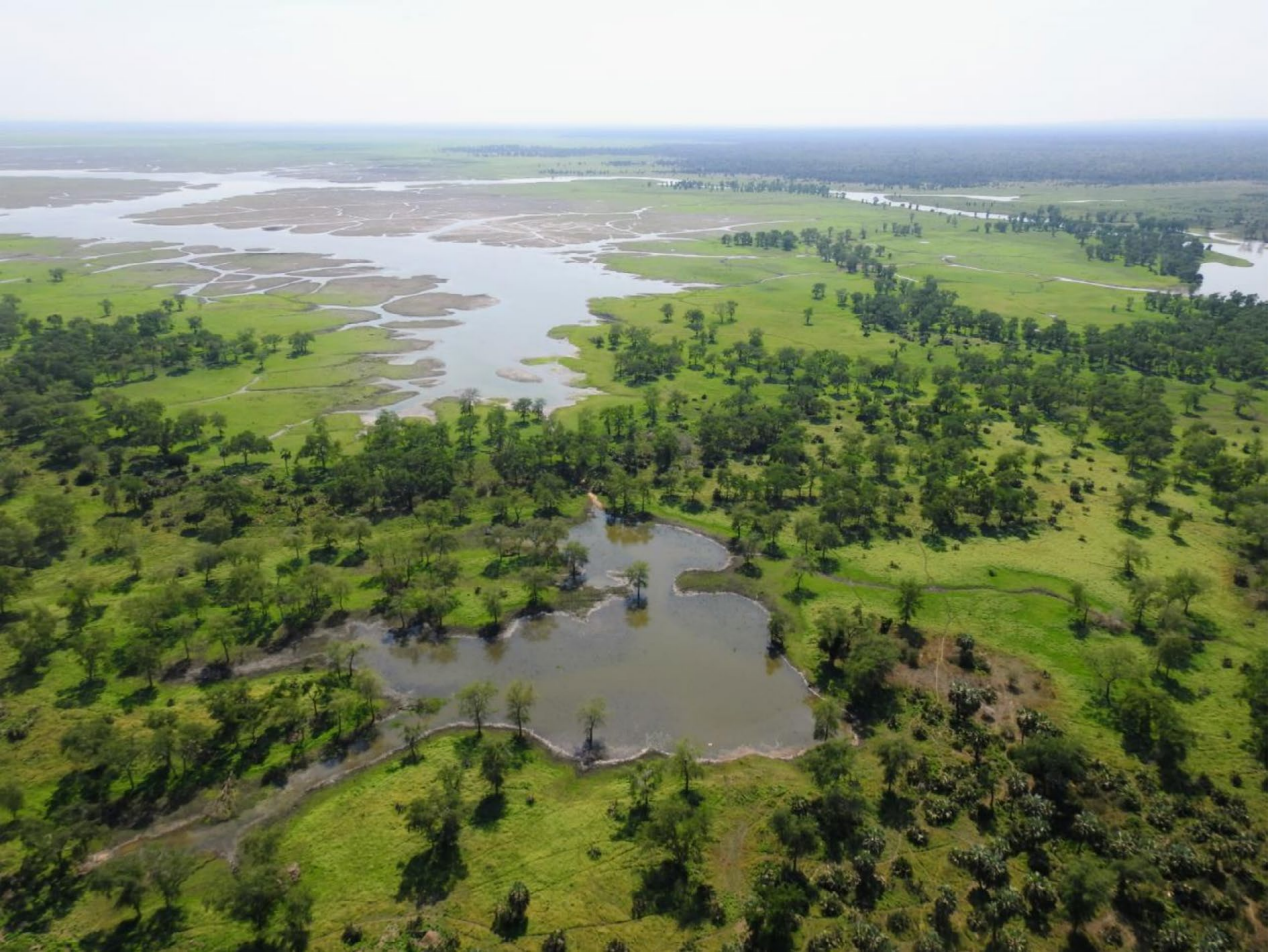
An aerial view of Gorongosa National Park at the edge of Lake Urema showing floodplain grasslands and woodlands. The park is characterized by heterogeneous and dynamic environments, with a lake at its core that floods extensively during the rainy season. The photograph shows flooded areas near the southern edge of the lake (Photo R. Bobe).

Located at the southern terminus of the East African Rift System, Gorongosa hosts heterogeneous environments and plant communities ranging from rainforests on Mount Gorongosa to floodplain grasslands surrounding Lake Urema at the heart of the park (Stalmans and Beilfuss 2008). The extent of the lake itself fluctuates significantly between the dry (April–November) and wet (November–March) seasons. This diverse environmental and ecological setting (Figure 1) presents an ideal context to study how primates adapt to highly seasonal, dynamic, and heterogeneous ecosystems, as illustrated in the articles in this special issue, *Primate adaptations in a seasonal and heterogeneous African ecosystem*.

## 2 Brief History of Gorongosa National Park

The Gorongosa region was first proclaimed as a game reserve in 1921, and the park was officially established as a protected area in 1960. During the 1960s and early 1970s, extensive studies of the park's fauna, flora, and ecology were carried out by Ken Tinley, who published his observations and insights as a doctoral dissertation (Tinley 1977), re‐edited as a book (Tinley 2020). Until the mid‐1970s, the main predators in the park were lions (with about 200 individuals), leopards (which at that time occurred throughout the entire Gorongosa‐Cheringoma area), and wild dogs, which ranged across the savanna and grassland areas. Buffalo were the most abundant mammals, followed by wildebeest, hippopotamus, waterbuck, zebra, and elephant (Tinley 1977). Some large mammalian herbivores had become locally extinct by 1970, including the white rhinoceros, the black rhinoceros, roan antelope, and tsessebe (Tinley 1977). But the biggest biodiversity losses occurred during the Mozambique Civil War of 1977–1992, which devastated wildlife as well as human communities throughout the country. According to some estimates, GNP lost nearly 90% of the large mammal populations (Stalmans et al. 2019). These losses included most of the mammalian carnivores: only a handful of lions were left after the Civil War, while hyenas, leopards, and wild dogs had all but disappeared. Since 2007, the Gorongosa Restoration Project (now called the Gorongosa Project), a public‐private partnership, has led to the successful recovery of the Gorongosa ecosystem, with lions now numbering more than 200 in the park, while wild dogs, hyenas, and leopards have been reintroduced and their numbers are growing (Bouley et al. 2021, 2018; Stalmans et al. 2019; Stalmans and Peel 2024). Today, Gorongosa represents a dynamic, recovering ecosystem (Pringle 2017; Pringle and Gonçalves 2022; Stalmans et al. 2019).

**FIGURE 2:**
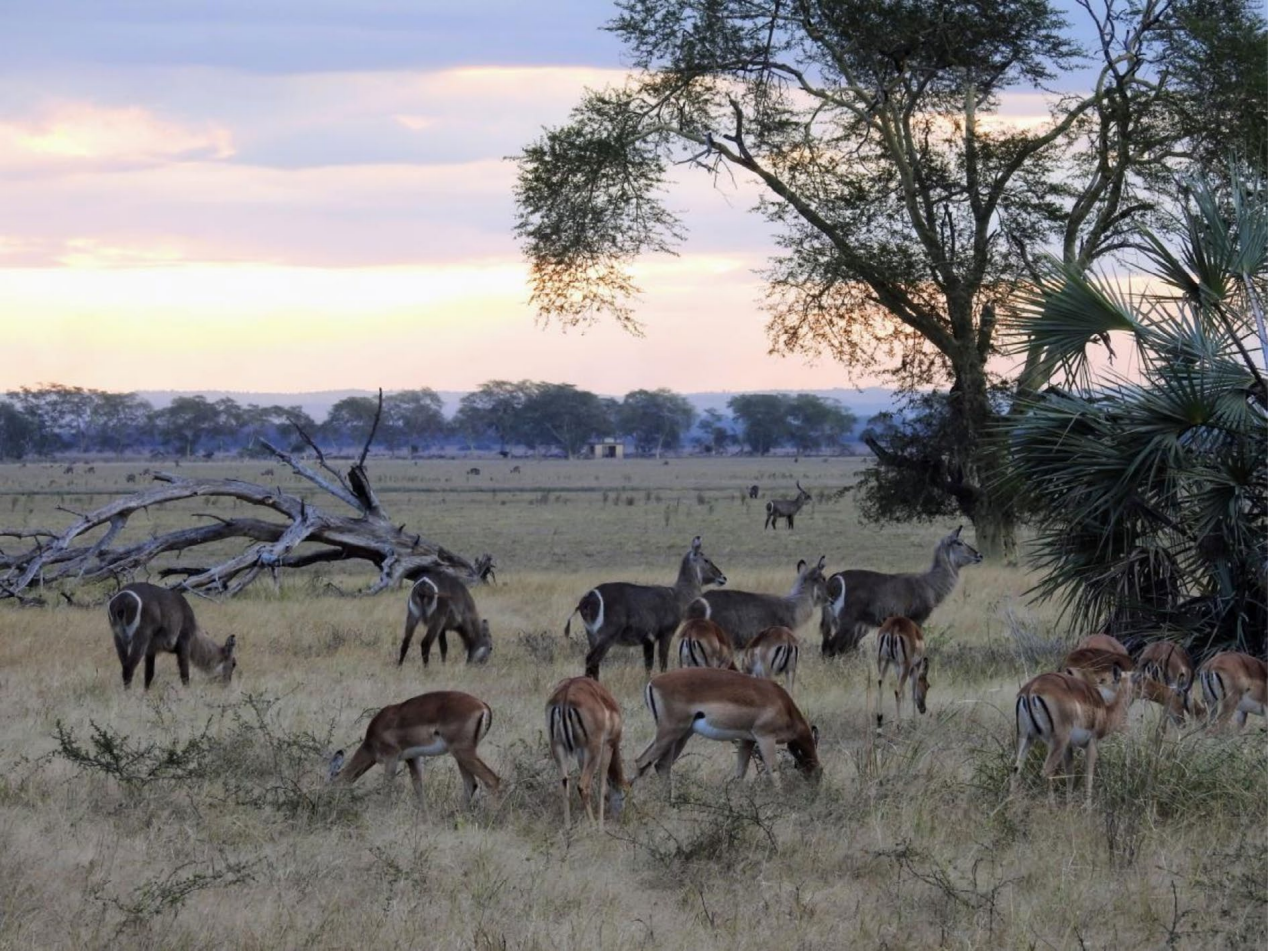
The two most common antelopes in Gorongosa are waterbuck and impala, pictured here in the Urema floodplain. The last aerial census of the park in 2024 counted 110,513 larger mammals. The Lion House, which researchers use as a vantage point to observe animals in the floodplain, is visible in the distance near the center of the photograph (Photo R. Bobe).

Results from the 2024 aerial census highlight the remarkable recovery and current composition of the large mammal community in the park (Stalmans and Peel 2024). Waterbuck ( *Kobus ellipsiprymnus* ) are the most abundant species (Figure 2), with an estimated 65,332 individuals, followed by impala ( *Aepyceros melampus* , 16,291 individuals), warthog ( *Phacochoerus africanus* , 4663 individuals), nyala ( *Tragelaphus angasii* , 4199 individuals), reedbuck ( *Redunca arundinum* , 4019 individuals), greater kudu ( *Tragelaphus strepsiceros* , 2781 individuals), and oribi ( *Ourebia ourebi* , 2000 individuals). Although baboons were not counted individually, the 229 troops documented suggest they may be the second or third most abundant mammal in the park, after waterbuck and impala. Independent camera‐trap data indicates that baboons are the most frequently detected of all mammals in the sectors of the park south of Lake Urema, whereas vervets, samangos, and bushbabies appear at lower detection rates among primates (Gaynor et al. 2021). The 2024 aerial census also counted 2748 crocodiles through direct visual identification, clearly a minimum number of the total in the park (Stalmans and Peel 2024). In addition to the aerial censuses, the park has a Biodiversity Exploration Program that keeps a growing Biodiversity Database that includes all living plants and animals (Stalmans and Naskrecki 2019), and currently has records of 8032 species in the park, with 83 described taxa that are new to science (https://gorongosa.org/map‐of‐life/) (P. Naskrecki, pers.com.).

## 3 The Gorongosa Ecosystem

In its current form, GNP covers an area of ~4000 km^2^, surrounded by the 5400 km^2^ Sustainable Development Zone that mediates interactions between protected wildlife and local communities (Pringle and Gonçalves 2022). The northern boundary of the park is located just south of the Zambezi River, while the Púnguè River marks the southern border of the park. Mount Gorongosa, with a peak at 1863 m above sea level, dominates the western part of the park, while the Cheringoma Plateau marks the eastern boundaries. Mount Gorongosa is key to the ecosystem as it captures the rainfall that flows into the Urema Graben and provides a critical year‐round source of fresh water for plants and animals on the Rift Valley floor. Mount Gorongosa receives more than 2000 mm of rainfall per annum, which sustains montane rainforests and gives rise to perennial rivers flowing into Lake Urema. East of the mountain lies the Midland region, dominated by miombo woodlands with tall *Brachystegia* trees. The Rift Valley floor receives 840 mm of rain and is dominated by seasonally flooded grasslands, with patches of tall acacia woodlands, mopane, combretum savannas, sand forests, and a network of termite mounds hosting patches of thick vegetation and tall trees (Tinley 1977). Rainfall, however, is highly variable from year to year, ranging, for example, from 1200 mm in 2014 to only 688 mm in 2015 (Daskin et al. 2023). This spatial and temporal variation in precipitation shapes the ecological complexity that defines the Gorongosa ecosystem.

Lake Urema is the hydrogeographic centerpiece of Gorongosa, and its extent fluctuates dramatically across seasons and years. In the 1960s and 1970s, the surface area of Lake Urema varied from about 10 km^2^ in the dry season to 200 km^2^ in the wet season (Tinley 1977). Satellite imagery has shown that in the interval from 1979 to 2000, the extent of the lake ranged from 17.4 km^2^ to 104 km^2^ (Böhme et al. 2006). In a typical year, the lake levels rise by up to 3 m, inundating large portions of the surrounding floodplain (Böhme 2005; Böhme et al. 2006). In some years, upwards of 780 km^2^ of Rift Valley floor around Lake Urema may be submerged (Walker et al. 2023). Tropical cyclones occur on average twice per year in Mozambique (Cabral et al. 2017; Kolstad 2021); they amplify these hydrological fluctuations and cause widespread environmental disruption (Beardmore‐Herd et al. 2025; Walker et al. 2023). Thus, a major feature of the ecology of the park is the cyclical expansion and contraction of Lake Urema. These pulses of expansion and contraction of the lake waters as well as the flooding and contraction of the major rivers and pans constitute a key driver of the annual cycle of life in the park (Tinley 1977). The floodplain surrounding the lake supports a high concentration of grazing ungulates, including waterbuck, reedbuck, oribi, and warthog, with diverse food resources concentrated close to the edge of the flood waters, while the woodlands away from flooded areas support browsing ungulates like nyala and kudu. Impalas are abundant in the ecotones (Stalmans and Peel 2024).

Ecological heterogeneity is further enhanced by the network of termitaria produced by fungus‐farming termites of the genus *Macrotermes*. These termite mounds concentrate nutrients and moisture, resulting in elevated dense patches of vegetation with tall trees that function as ecological islands at times of flooding. These ecological islands are preferentially used by some antelopes like bushbuck, nyala, and kudu (Daskin et al. 2023) as well as primates like baboons and vervets. Termite mounds also serve as fire refugia, shielding vegetation and animals during the annual grassland fires that sweep through the Rift Valley (Tinley 1977).

In some key aspects, the Gorongosa modern ecosystem resonates with the paleoenvironmental reconstructions of early hominin sites across the East African Rift, where much of the fossil record of early hominins has been found, e.g., the Middle Awash (Haile‐Selassie and WoldeGabriel 2009; White et al. 2009), Dikika (Wynn et al. 2006), Hadar (Campisano et al. 2022), the lower Omo Valley (Negash et al. 2024), the Lake Turkana region (Bobe et al. 2022), Tugen Hills (Kingston et al. 2007; Pickford and Senut 2001), and others. Paleoenvironmental reconstructions of these Rift Valley sites consistently point to complex, heterogeneous, and dynamic environments consisting of vegetation mosaics of forests, woodlands, wetlands, and grasslands in various proportions. These paleontological sites are also associated with past conditions indicating abundant fresh water in lakes, pans, rivers, or deltaic environments. Fossil hominins are also typically found alongside a rich record of mammalian species with a high diversity of ungulates, as well as other primates. These ecological conditions in herbivore‐rich ecosystems recall key aspects of modern‐day GNP.

## 4 The Primates of Gorongosa National Park

Gorongosa currently supports five primate species: baboons ( *Papio ursinus griseipes* ), vervet monkeys ( *Chlorocebus pygerythrus pygerythrus* ), samango or Stairs' white‐collared monkeys ( *Cercopithecus mitis erythrarchus* ), and two species of nocturnal galagids—the large‐eared greater galago ( *Otolemur crassicaudatus* ) and Mozambique dwarf galago ( *Paragalago granti* ). The southern lesser galago, *Galago moholi* , occurred in the vicinity of the park in historical times but has not been directly documented in decades. In the sections that follow, we briefly review the occurrence and ecological context of each taxon in Gorongosa as a baseline for future research.

### 4.1 *Papio ursinus griseipes*

**FIGURE 3:**
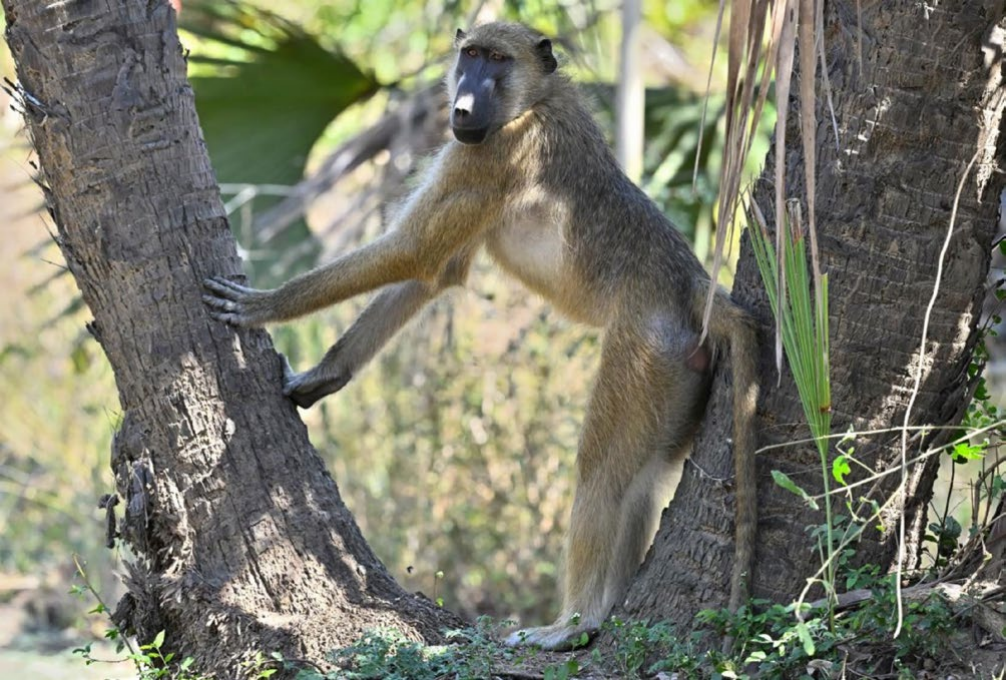
Gorongosa baboons are classified as *Papio ursinus griseipes* (grayfoot chacma), but exhibit physical characteristics of both chacma and yellow baboons. Genetic evidence indicates some degree of hybridization between *P. ursinus* and *P. cynocephalus* (Photo J. Fernandes).

Baboons (genus *Papio*) occupy extensive regions of sub‐Saharan Africa and the southwest of the Arabian Peninsula. With diverse geographic variants, adjoining ranges, and frequent interbreeding, populations of the genus *Papio* are sometimes divided into subspecies of widely distributed *Papio hamadryas* , or, using the phylogenetic and ecological species concepts, into six distinct, sexually dimorphic species including *P. hamadryas* (hamadrayas baboons), *P. papio* (Guinea baboon), *P. anubis* (olive baboon), *P. cynocephalus* (yellow baboon), *P. kindae* (Kinda baboon), and *P. ursinus* (chacma baboon) (Fischer et al. 2019; Ossorio et al. 2025; Zinner et al. 2011; also https://www.iucnredlist.org/). It is clear, however, that hybridization occurs between some of these species across contact zones (Chiou 2017; Jolly et al. 2011; Rogers et al. 2019). Although the Gorongosa baboons are classified as chacma baboons, *Papio ursinus* , they belong to the distinct subspecies of grayfoot chacma, *Papio ursinus griseipes* , and have some phenotypic as well as genomic affinities with yellow baboons, reflecting a complex evolutionary history (Figure 3) (Caldon et al. 2025; Ferreira da Silva et al. 2025; Martínez et al. 2019; Santander et al. 2022). Genetic and paleontological data indicate that the last common ancestor of modern baboons originated in the Pliocene or early Pleistocene in southeastern Africa (Burrell 2009; Wildman et al. 2004; Zinner et al. 2011) in regions that may well have included the ancient Gorongosa ecosystem (see Figure 7.8 in Zinner et al. 2011). Baboons are the most abundant primates in GNP, as well as one of the most abundant mammals overall (Stalmans and Peel 2024). Camera‐trap studies demonstrate exceptionally high occupancy probabilities, with densities surpassing those in the Serengeti and Okavango ecosystems (Gaynor et al. 2021). These data underscore Gorongosa's significance as a population stronghold and hotspot for *Papio* in Africa.

### 4.2 *Chlorocebus pygerythrus pygerythrus*

**FIGURE 4:**
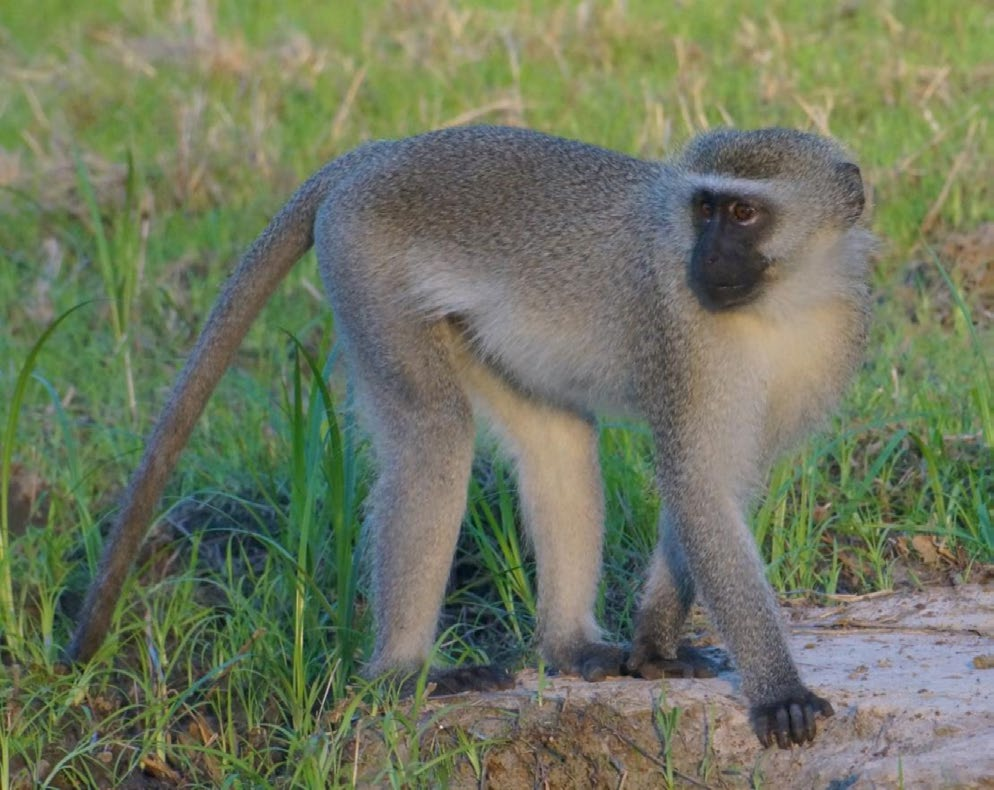
Vervets are abundant and widespread in parts of the Gorongosa ecosystem (Photo M. Beardmore‐Herd).

Vervets, or savanna monkeys, in the genus *Chlorocebus* occupy diverse environments across much of sub‐Saharan Africa, with the exception of Guineo‐Congolian rainforests and true deserts (Turner et al. 2018). Vervets are omnivorous and spend time foraging both arboreally and terrestrially (Isbell and Enstam Jaffe 2013). Their semi‐terrestrial adaptations are evident in several aspects of their skeletal anatomy, such as elongated radio‐ulnae and tibiae relative to humeral and femoral lengths (Anapol et al. 2005). These semi‐terrestrial adaptations led Benefit, McCrossin, and colleagues to regard vervets as a model for the ancestral cercopithecoids that evolved from the semi‐terrestrial Miocene genus *Victoriapithecus* (Benefit and McCrossin 1997; McCrossin et al. 1998). Molecular evidence indicates that the genus *Chlorocebus* originated in east/central Africa more than half a million years ago, with *C. pygerythrus* expanding southward around 265 ka (Turner et al. 2018). Fossil vervets are extremely rare, but specimens of *Chlorocebus* from the paleontological site of Asbole, Ethiopia, dated to 600 ka corroborate the genetic data that the genus had emerged early in the middle Pleistocene (Frost and Alemseged 2007). The vervet monkeys of Gorongosa NP (Figure 4) are considered a subspecies of *C. pygerythrus* , which inhabits diverse savanna woodlands and riverine forests from Ethiopia to South Africa (Turner et al. 2019). Locally, vervets are known as *macaco de cara preta* (Portuguese for black‐faced monkey). Although these primates have long been studied in places like Amboseli in Kenya (Cheney et al. 1981; Cheney and Seyfarth 1983; Isbell et al. 1990) and in South Africa (Henzi 2008; Henzi and Lucas 1980), research in Gorongosa represents the first systematic studies of vervets in Mozambique (Beardmore‐Herd et al. 2025).

### 4.3 *Cercopithecus mitis erythrarchus*

**FIGURE 5:**
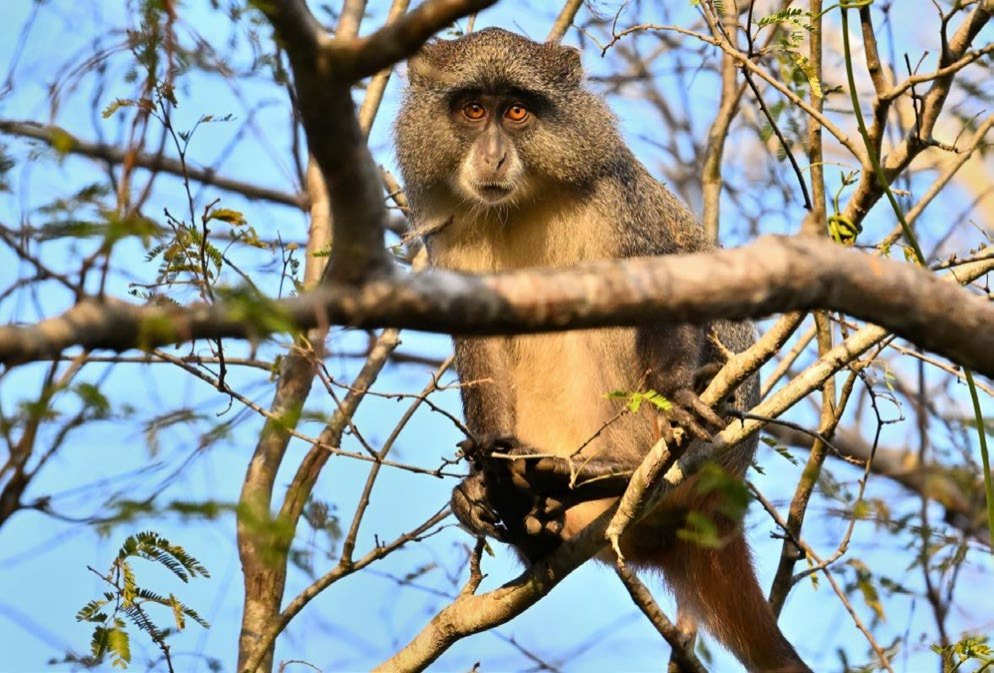
Samango monkeys are locally abundant in some of the park's woodlands, and studies of their ecology, taxonomy, and genetics are a high priority for future primatological research at Gorongosa (Photo J. Fernandes).

In his 1977 monograph, Tinley describes the samango monkey as occurring in all forest areas of the Gorongosa ecosystem (Tinley 1977). In the Rift Valley, the samango monkey “uses the tall riverine thickets as well as venturing out from larger forest areas to feed or pass through the archipelagos of termitaria thickets” (Tinley 2020, 249). Up until the 1970s, the most important predator of the samangos was leopards, “which abound throughout the entire Gorongosa‐Cheringoma area” (Tinley 2020). At least five troops of samango monkeys live within the boundaries of the park (Stalmans and Peel 2020). Although locally referred to as samango monkeys (Figure 5), the Gorongosa species may also be called Stairs's white‐collared monkey following the nomenclature of the IUCN (https://www.iucnredlist.org/species/136887/196010434) as well as descriptions in Mammals of Africa (Volume II: Primates) (Butynski et al. 2013; Lawes et al. 2013). Despite their ecological importance, these primates have yet to be well studied in Gorongosa, but will be the focus of research in the near future.

### 4.4 *Otolemur crassicaudatus*

**FIGURE 6:**
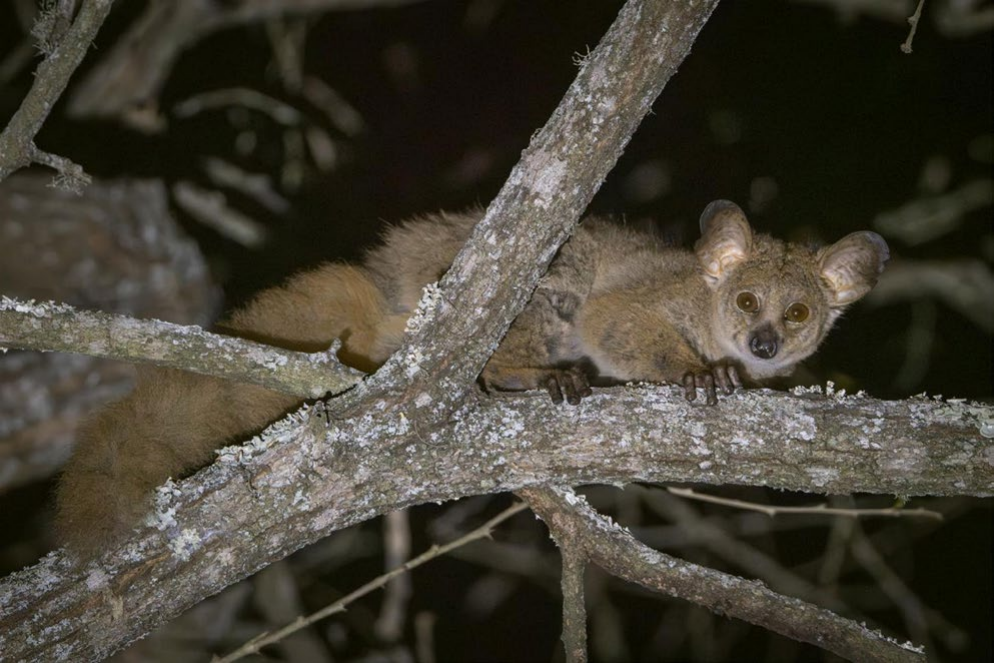
The most commonly seen strepsirrhine in Gorongosa is the large‐eared greater galago (also called the thick‐tailed bushbaby), *Otolemur crassicaudatus* (Photo P. Naskrecki).

The nocturnal strepsirrhines of the family Galagidae are often said to be the least known of all primate clades (Nekaris and Bearder 2011; Penna and Pozzi 2024), and there is not a single study of galagid behavior, abundance, or ecology in Mozambique. Nevertheless, at least two species of galagids are known to occur in Gorongosa NP: the large‐eared greater galago (or thick‐tailed bushbaby) *Otolemur crassicaudatus* and the dwarf galago *Paragalago granti* . *Otolemur* is commonly seen during night drives (Figure 6) and is heard almost nightly in the park's headquarters of Chitengo. Recent studies of this species have relied on bioacoustics, genomics, and body mass data from locations in Kenya, Tanzania, and South Africa (Leigh et al. 2024; Pozzi et al. 2019). The loud calls of *O. crassicaudatus* provide an example of the recognition concept of species, a concept elaborated by Paterson (Paterson 1985). The species of *Otolemur* are described as trailing callers. At Lajuma Research Centre in northern South Africa, thick‐tailed galago male body mass has been estimated to be about 1.24 kg, whereas females weigh about 1.03 kg. These are the largest galagos (Bearder and Svoboda 2013). In this issue, Osório and Veracini note that the National Museum of Natural History and Science in Lisbon, Portugal, holds 8 specimens collected from the Gorongosa region in 1948 and 1955. A thorough study of these specimens would add significantly to our knowledge of the thick‐tailed galagos from Gorongosa.

### 4.5 *Paragalago granti*

**FIGURE 7:**
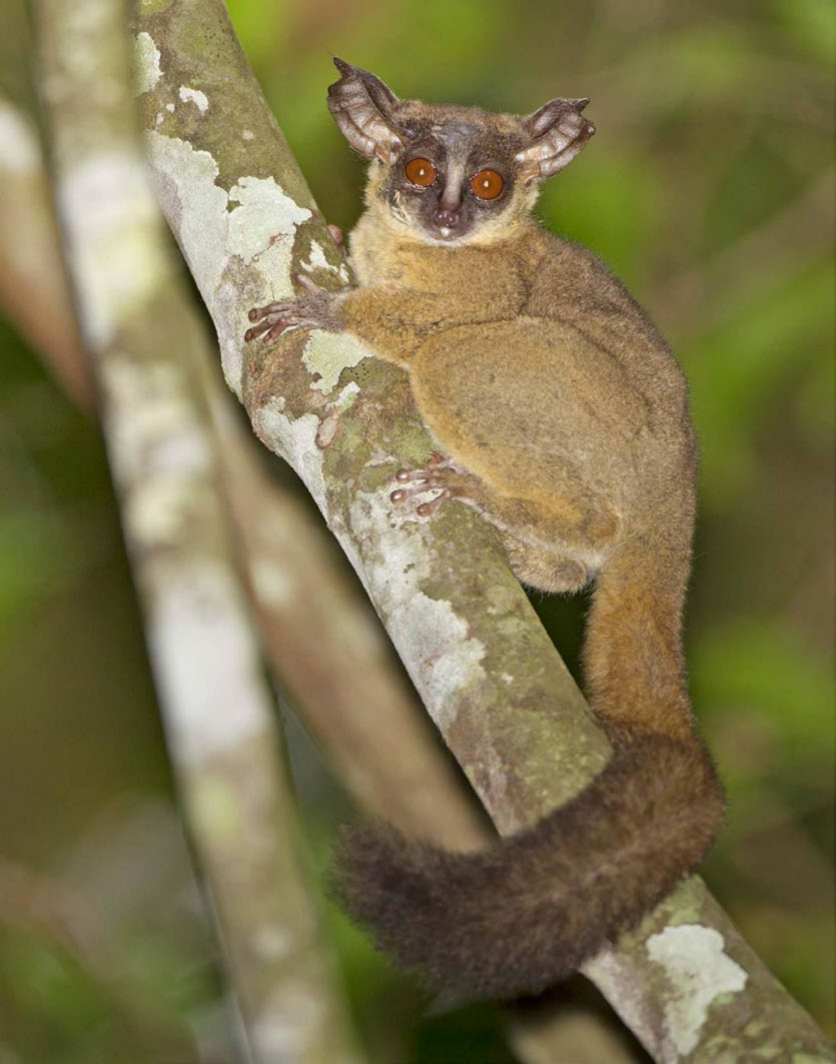
Grant's dwarf galago, *Paragalago granti* , is found in woodlands and coastal forest mosaics. This photo is from the Inhamitanga Forest near the Zambezi River (Photo Tertius A. Gous).

The eastern dwarf galagos have been assigned to their own genus, *Paragalago*, on the basis of distinct vocalizations, morphology, and DNA sequence data (Masters et al. 2017; Penna and Pozzi 2024). Grant's dwarf galago, *Paragalago granti* , is a species distributed across Zambezian woodlands and coastal forest mosaics from Tanzania to South Africa on the east side of the East African Rift (de Jong et al. 2019). The geographic distribution of this species in the woodlands and forests of eastern Africa offers a prime example of Jonathan Kingdon's recognition of a “Zanj” biogeographic center of endemism spreading from the coastal regions of southern Somalia to Mozambique (Groves 2015; Kingdon 1971). This species is known to occur in Gorongosa, as it is frequently heard and sometimes seen by park rangers and visitors, but the species has not yet been studied in any detail. From a few available studies elsewhere, the body mass of this species is about 134 g, and individuals sleep in groups of 4–5 adults and offspring (Nekaris and Bearder 2011). Clearly, the dwarf galagos of Gorongosa (Figure 7) provide a major opportunity for the long‐term study of their ecology, morphology, and evolutionary relationships.

### 4.6 *Galago moholi*

The southern lesser galago, *Galago moholi* , may have been present in the Gorongosa region in historical times as suggested by two specimens collected near Vila Gorongosa in 1955 (Queirós Neves 2020). However, there are no confirmed records of this species occurring in the park in recent times, and the known geographic distribution of the southern lesser galago includes areas mostly to the west of the park (Pullen and Bearder 2013). *Galago moholi* is a repetitive caller (Pullen et al. 2000), and its cries are quite distinctive from *O. crassicaudatus* and *P. granti* . Considering the lack of recent records, bioacoustic and visual surveys in the drier areas of the park (open woodland, savanna, bush, and forest fringe) are needed to confirm if the species is present in the Gorongosa ecosystem.

## 5 Highlights of the Papers From This Volume

At a time of significant climatic and environmental disruptions worldwide, it has become imperative to study how natural populations respond to extreme and unpredictable events. On 15 March 2019, Cyclone Idai made landfall in central Mozambique, passing directly over Gorongosa NP. This cyclone has been characterized as the most powerful on record in the southern hemisphere (Charrua et al. 2021) and led to widespread flooding in central Mozambique, including the Gorongosa region. Beardmore‐Herd and colleagues were able to track the distribution of baboons and vervets before and after the passage of the cyclone (Beardmore‐Herd et al. 2025). This opportunistic study relied on camera‐trap data to document that during the first month after the cyclone, baboons were able to change their spatial distribution and were more frequently detected in areas less affected by flooding. The sample from vervet monkey detections was too small to reach reliable conclusions, but primate abundances overall did not decrease before and after the cyclone.

Hammond et al. (2025) explored terrestriality in Gorongosa baboons by means of two sets of camera trap grids, one within GNP and the other just outside the park (Levas Flores forestry concession). Within the boundaries of GNP, baboons accounted for 15% of all detections in camera traps, whereas in the miombo woodlands of the forestry concession they accounted for 8% of all animal detections. Lions were detected in the grid within the park, while leopards were detected in the grid outside the park. After considering several variables, the authors conclude that baboons tend to be more terrestrial during the late stages of the dry season, when arboreal resources become scarce and the need to procure water at ground level increases.

The Gorongosa baboons also illustrate another key aspect of their adaptive repertoire: regional variation in behavioral practices. Biro and colleagues surveyed an area of 300 km^2^ with camera traps across Gorongosa NP, an area that encompasses the range of 60 baboon troops. The authors documented the frequency of baboon bark‐stripping of *Acacia robusta* trees and found that this behavior is absent in some areas but prevalent in others (the western part of the range), suggesting that this behavior may be socially transmitted (Biro et al. 2025).

The paper by Lewis‐Bevan et al. (2025b) presents the first empirical data on Gorongosa baboon sleeping sites and shows that these primates are quite flexible in their choices of sites and use different locations throughout their home range. Two troops of baboons were tracked observationally on the ground and with the use of GPS collars, with data collected from 2017 to 2019, with two baboons collared in each troop, for a total of 1160 days and nights of data. These data were collected at a time when GNP still had a relatively low predator density, which by 2022–2025 had increased significantly. The authors also showed that Gorongosa baboons tend to sleep close to the last resources they encountered on the day (areas of interest), usually preferring continuous patches of dense woodland to more sparse patches of trees. This study provides a baseline for further research as predator populations continue to increase. Alongside the other contributions in this volume, it constitutes a foundational basis for future primatological research in GNP.

Farassi et al. (2025) focus on object manipulation in baboons, which may help us understand the ecological pressures related to the emergence of tool use in other primates. The authors provide an extensive discussion of our current understanding of tool use and object manipulation across the primate order. Their study of 3 troops in Gorongosa resulted in 787 events involving object use. Mixed model logistic regression analysis of the data showed that object use was more likely in open forests than in the plains, more common arboreally than terrestrially, and that the frequency of this behavior decreases with the age of the individual from the juvenile stages to adulthood.

Muschinski assessed another aspect of potential regional variation in baboons: greeting behaviors among males (Muschinski 2025). She analyzed video footage of male–male rates and intensity of physical contact as well as visual reciprocity in Gorongosa baboons, and then made comparisons with those of baboons from other parts of Africa. Muschinski found that Gorongosa male greeting behavior is similar to that in populations of yellow, olive, and hamadrayas baboons, but differs from those of Guinea baboons. The author also discusses the social functions of these greeting behaviors.

The taxonomic affinities of Gorongosa baboons within the genus *Papio* have been a matter of some discussion. Gorongosa populations are usually classified within chacma baboons, *Papio ursinus* , but show several phenotypic characteristics of yellow baboons, *Papio cynocephalus* (Ferreira da Silva et al. 2025; Martínez et al. 2019; Santander et al. 2022). To address this issue with genomic data, Caldon and colleagues (Caldon et al. 2025) analyzed uniparental genomic markers (mitochondrial DNA and Y chromosome) as well as autosomal variants of baboons from Gorongosa (including the Catapu area) and found evidence of introgression from eastern Tanzanian yellow baboons. They also estimated that the last common ancestor of Gorongosa, Zambia, and Tanzania yellow baboons lived about 1 million years ago.

Traditionally, museum specimens have played a key role in the taxonomic studies of galagos, with an emphasis on teeth, skeletal features, and pelage. In this special issue, Osorio and Veracini offer a valuable account of historical collections housed at the National Museum of Natural History and Science in Lisbon, Portugal (Osório and Veracini 2025). The collection derives from expeditions in 1948 and 1955 to parts of Mozambique that included what would become Gorongosa National Park. The authors offer a detailed account of the historical context in which these valuable collections took place.

## 6 Conclusions and Future Directions

The papers in this special issue are the first wave of primatological research in Mozambique and demonstrate that the Gorongosa ecosystem is fertile ground for the growth of primate studies in southeastern Africa. Ongoing research by PhD students is focused on primate socioecology (Rabajoli and Carvalho 2025) and on the relationship between primates and other mammals such as species of ungulates, as well as between primates and aquatic predators such as crocodiles. Besides studies of baboon and vervet behavior and ecology, we expect future work to focus on samango monkeys and galagos, and to expand to these species the studies of primate genetics and genomics (Ferreira da Silva et al. 2025). Bioacoustic research methods will be needed to ascertain the diversity, abundance, and distribution of nocturnal primates. This research is embedded in a program of teaching and mentoring the first generation of Mozambican primatologists.

## Author Contributions

**Susana Carvalho:** conceptualization, writing – original draft, writing – review and editing, supervision, funding acquisition, project administration. **Robert L. Anemone:** writing – review and editing. **João d'Oliveira Coelho:** writing – review and editing, visualization, supervision. **René Bobe:** conceptualization, funding acquisition, writing – original draft, supervision.

## Conflicts of Interest

The authors declare no conflicts of interest.

## Data Availability

Data sharing not applicable to this article as no datasets were generated or analysed during the current study.
